# Investigating Potential Molecular Mechanisms of Carcinogenesis and Genes as Biomarkers for Prognosis of Gastric Cancer Based on Integrated Bioinformatics Analysis

**DOI:** 10.1007/s12253-018-0523-4

**Published:** 2018-11-14

**Authors:** Yi Zhu, Xiangwei Sun, Ji Lin, Teming Zhang, Xin Liu, Xian Shen

**Affiliations:** 1 The Second Affiliated Hospital and Yuying Children’s Hospital of Wenzhou Medical University, No. 109, Xueyuan West Road, Lucheng District, Wenzhou, Zhejiang, 325000 People’s Republic of China; 2 First Affiliated Hospital of Wenzhou Medical University, Wenzhou, China

**Keywords:** Gastric cancer, GEO data, lncRNAs, Integrated bioinformatics, Regulatory network

## Abstract

Gastric cancer, as the fifth most common malignancy worldwide, is a deadly disease afflicting nearly a million people. Researchers have devoted much to study the mechanisms of carcinogenesis and progression, but the exact information of tumor initiation and progression is remained largely unknown. Here, we hypothesized several differentially expressed genes and possible pathways by employing integrated bioinformatics analysis. We fully analyzed four gastric cancer-related microarray datasets to screen differentially expressed mRNAs (DEMs), miRNAs (DEMis) and lncRNAs (DELs). The functional enrichment analysis was deeply construed, PPI network and ceRNA regulatory network were constructed to investigate potential mechanisms of tumorigenesis and progression. Furthermore, survival analysis was performed to identify critical lncRNAs that may significantly affect pathogenesis of gastric cancer. QRT-PCR was applied to verify our result. We identified two hub subnetworks that may explain the progression, metastasis and poor prognosis of gastric cancer. Meanwhile, several potential significant lncRNAs were identified. In summary, we ascertained several significantly changed KEGG pathways in the tumor initiation and progression. We also hypothesized several lncRNAs that contribute to poor prognosis of gastric cancer via integrated bioinformatics, which deserve further investigation.

## Introduction

Gastric cancer occupies the fifth most incident and the third cancer-associated deaths worldwide, afflicting nearly a million people and leading to 723,000 deaths annually [1]. About half of the disease attacks Eastern Asia, particularly in China, Japan and South Korea [1]. However, the mortality of gastric cancer in South America and Eastern Europe is higher, because routine screening of early gastric cancers is well performed in Japan and South Korea [2, 3]. The initiation and progression of gastric cancer involve enormous changes in gene regulation, mRNA, miRNA and lncRNA included. Biomarkers which provide information for early diagnosis and prognosis can be significant in treating gastric cancer.

In the present study, we fully exploited datasets from the GEO online database to compare the gene expression change between tumor samples and para-cancer tissues by analyzing four microarray datasets [4, 5]. Overlapped differentially expressed miRNA in GSE28700 and GSE99415 were selected for further mRNA and lncRNA prediction. GO and KEGG enrichment analysis for DEMs and PPI network construction were performed to explore insightful pathways for analyzing the miscellaneous information. Screening of hub sub network was also applied to minimize the range of DEMs. Furthermore, we applied ceRNA and survival analysis to investigate significant lncRNAs that play critical roles in gastric cancer initiation and progression. Furthermore, we validated the result via qRT-PCR. Our result provides an insightful understanding of gastric cancer pathogenesis, tumorigenesis and progression, and identified hub lncRNAs may serve as biomarkers for gastric cancer prognosis.

## Materials and Methods

Microarray data. We searched the GEO online database to obtain the microarray for analysis of gastric cancer. Series matrix files of microarray data: GSE54129, GSE28700, GSE99415 and GSE99416 were selected for following analysis [4, 5]. A total of 111 surgically resected gastric cancer tissues and 21 matched normal tissues were achievable in GSE54129, based on GPL570 Affymetrix Human Genome U133 Plus 2.0 Array. GSE28700 and GSE99415 were for miRNA analysis, there were 22 gastric cancer tissues and 22 matched normal tissues, 6 gastric cancer and 6 matched normal tissues in them respectively, based on GPL9081 Agilent-016436 Human miRNA Microarray 1.0 G4472A and GPL18058 Exiqon miRCURY LNA microRNA array, 7th generation. Similarly, we obtained 6 gastric tumor tissues and 6 matched normal tissues from GSE99416, based on GPL16956 Agilent-045997 Arraystar human lncRNA microarray V3.

Data processing. Background correction and quartile data normalization of the original microarray data were performed for each series by applying the robust multi-array average algorithm. Probe names were annotated gene symbols according to annotation files respectively. After filtering those probes without corresponding gene symbols, the average value of gene symbols with multiple probes were calculated. We performed comparison between selected gastric tumor tissues and their matched normal samples by using R software limma package [6]. The mRNA with |log2(fold change) | > 1.585 and *p* value <0.05 were regarded as differentially expressed mRNA (DEMs), miRNAs and lncRNAs with |log2(fold change) | > 1 and *p* value <0.05 were considered as DEMis and DELs. To further intensify its reliability, we only analyzed the miRNA with same expression change in GSE28700 and GSE99415.

GO and KEGG analysis. Gene Ontology, including three sections: biological process (BP), cellular component (CC) and molecular function (MF), is helpful for researchers to comprehensively understand biological meaning behind colossal list of genes. Kyoto Encyclopedia of Genes and Genomes (KEGG) is an insightful approach to understand high-level functions and utilities of the biological system, like tissues and cells, from molecular-level information generated by high-throughput sequencing and other technologies. Database for Annotation, Visualization and Integrated Discovery (DAVID, https://david.ncifcrf.gov/) online database was applied to investigate enriched pathway and process introduced by DEMs [7]. Terms with a *p* value <0.05 were considered to be significant.

PPI network construction and hub subnetwork screening. Protein-protein interactions were available in Search Tool for the Retrieval of Interacting (STRING, https://string-db.org/) online database. Predictions with combined score ≥ 0.7 in STRING was chosen for further visualization. The PPI network was visualized by applying Cytoscape (http://www.cytoscape.org/). To minimize the range of DEMs and enhance the reliability of our result, we applied PPI network complex for further discussion, two hub subnetworks were screened by applying MCODE app [8]. Function enrichment analysis of these selected genes was also performed.

ceRNA network construction. TargetScanHuman 7.2 (http://www.targetscan.org/vert_72/) and LncBase Predicted v.2 (http://carolina.imis.athena-innovation.gr/diana_tools/web/index.php?r=lncbasev2%2Findex-predicted) were online database for the prediction of interactions between miRNA and mRNA, interactions between miRNA and lncRNA, respectively. Predictions with Cumulative weighted context++ score < −0.4 in TargetScanHuman were selected, as well as those with a miTG-score > 0.7 in LncBase Predicted v.2. The ceRNA network was visualized by using Cytoscape. Kaplan–Meier survival analysis was performed for several lncRNAs with gastric cancer data in TCGA (The cancer genome atlas, https://cancergenome.nih.gov/).

Patients and tissue specimens. In our study, we collected 11 gastric cancer tissues from patients who received tumor resection at the Department of Abdominal Surgery. All selected patients were confirmed by histology. Our study was approved by the Ethical Committee and Institutional Review Board of The Second Affiliated Hospital and Yuying Children’s Hospital of Wenzhou Medical University. 11 matched para-cancerous tissues were also collected. Immediately following resection, gastric cancer tissues and corresponding normal tissues were frozen in liquid nitrogen and stored at −80 °C before using. Each patient participated in this study provided informed consent.

**Fig. 1:**
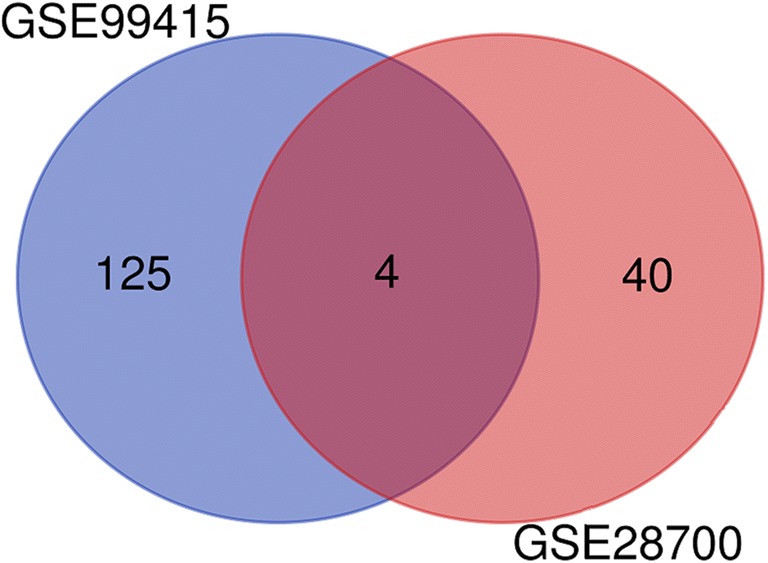
Identification of overlapping DEMis. Venn diagram of 4 overlapping DEMis in GSE28700 and GSE99415

RNA extraction and quantitative real-time PCR. Trizol reagent (Cat.No.15596–026, Invitrogen) was applied to extract total RNA from the collected tissues according to the manufacturer’s protocol. RNase-free DNase I (Cat.No.2270A, Takara) was used to dislodge genome from the samples collected. Before qRT-PCR, each RNA sample was reversed transcribed into cDNAs applying PrimeScript™ RT reagent Kit (Cat.No.RR037A, Takara). The qRT-PCR was conducted to measure the levels of mRNAs using the comparative Ct method according to manufacturer’s protocol (Cat.No.RR420A, Takara). Table 1 lists the primer sequences for PCR.

**Table 1:** Primer sequences of PCR

cDNA	Forward primer (5′-3′)	Reverse primer (5′-3′)
SNHG4	AGTAGGGCATCCTTCACCCA	TGGTATCTTCAACGGGATCGT
LINC00523	AGTAGATACCACAGGCCCCC	GACCTCTGGGGAACATTGCT

## Result

### Identification of Differentially Expressed Genes

With the threshold of |log_2_(fold change) | > 1.585 and *p* value <0.05, we obtained 1867 dysregulated mRNAs in GSE54129, including 966 down-regulated and 901 up-regulated DEMs. Taking |log2(fold change) | > 1 and *p* value <0.05 as the cut-off point, a total of 44 dysregulated miRNAs was screened in GSE28700, 30 down-regulated and 14 up-regulated miRNAs included. Similarly, there were 129 differentially expressed miRNAs in GSE99415, contained 94 down-regulated and 35 up-regulated miRNAs. To intensify the reliability of result, the miRNAs with same expression change in GSE28700 and GSE99415 were selected as the DEMis for further analysis (Fig. 1). DEMis showed opposite expression were omitted. Three miRNAs were screened, including hsa-miR-133a, hsa-miR-375, hsa-miR-767-3p, which were all down-regulated. Meanwhile, we identified 4329 lncRNAs dysregulated in GSE99416 between the gastric cancer and matched adjacent non-tumorous tissues. The DELs contained 1974 up-regulated lncRNAs and 2355 down-regulated lncRNAs.

**Fig. 2:**
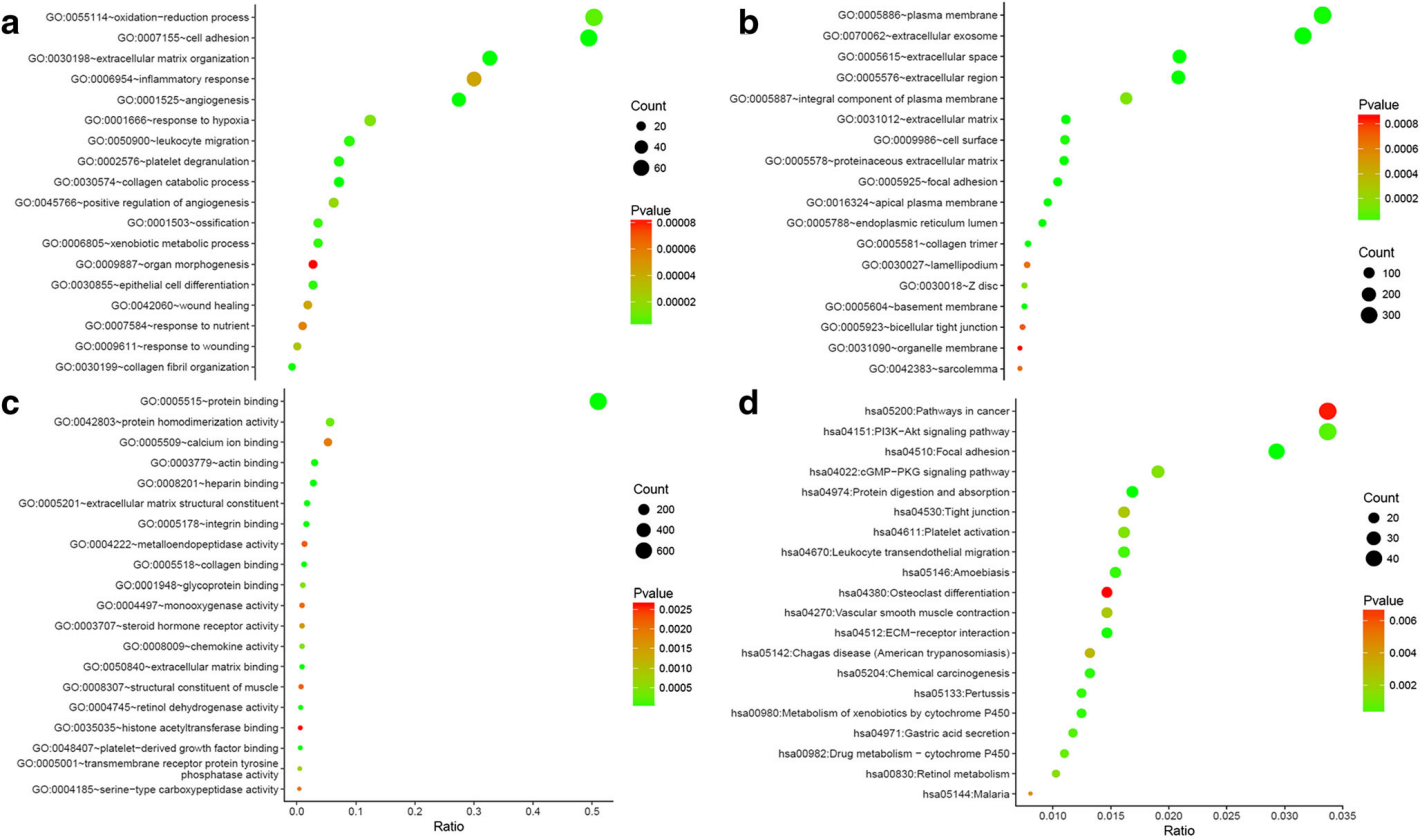
Functional enrichment of the DEMs. Notes: **a** Biological process (BP); **b** Cellular components (CC); **c** Cellular components (CC); **d** KEGG pathway analysis of DEMs

### Functional Enrichment Analysis

Figure 2 shows the top 20 terms of functional enrichment analysis result of identified DEMs by applying DAVID online database. The most important terms included in BP were extracellular matrix organization, angiogenesis, cell adhesion, endodermal cell differentiation and epithelial cell differentiation. The result was verified in the KEGG pathway analysis. DEMs were mainly involved in pathways in cancer, PI3K-Akt pathway, focal adhesion, ECM-receptor interaction and Metabolism of xenobiotics by cytochrome P450 in the level of KEGG pathway. Further, we performed KEGG pathway analysis with the up-regulated DEMs and down-regulated DEMs, respectively.

It is noteworthy that the up-regulated genes are mainly involved in focal adhesion, ECM-receptor interaction and PI3K-Akt signaling pathway. The down-regulated genes are mainly involved in pathways related to metabolism. Part of the KEGG analysis result was shown in Tables 2 and 3.

**Table 2:** KEGG pathway analysis for the down-regulated genes (top 10 terms)

KEGG pathway	KEGG entry	Count	Ratio	PValue
Metabolic pathways	hsa01100	84	0.115385	9.92E-09
Retinol metabolism	hsa00830	14	0.019231	6.13E-07
Gastric acid secretion	hsa04971	12	0.016484	7.49E-05
Metabolism of xenobiotics by cytochrome P450	hsa00980	12	0.016484	8.52E-05
Mucin type O-Glycan biosynthesis	hsa00512	8	0.010989	1.16E-04
Chemical carcinogenesis	hsa05204	12	0.016484	1.75E-04
Drug metabolism – cytochrome P450	hsa00982	11	0.01511	1.96E-04
Maturity onset diabetes of the young	hsa04950	7	0.009615	3.20E-04
Peroxisome	hsa04146	10	0.013736	0.003693
Fatty acid degradation	hsa00071	7	0.009615	0.00558

**Table 3:** KEGG pathway analysis for the up-regulated genes (top 10 terms)

KEGG pathway	KEGG entry	Count	Ratio	PValue
Focal adhesion	hsa04510	38	0.059375	1.88E-14
ECM-receptor interaction	hsa04512	20	0.03125	1.90E-09
PI3K-Akt signaling pathway	hsa04151	38	0.059375	8.56E-08
Pertussis	hsa05133	16	0.025	3.44E-07
Protein digestion and absorption	hsa04974	17	0.026563	5.50E-07
Amoebiasis	hsa05146	18	0.028125	1.54E-06
Platelet activation	hsa04611	20	0.03125	1.60E-06
Leukocyte transendothelial migration	hsa04670	18	0.028125	6.98E-06
Vascular smooth muscle contraction	hsa04270	18	0.028125	7.84E-06
Phagosome	hsa04145	20	0.03125	1.83E-05

PI3K-Akt pathway, activated by gene mutation and copy number, represents more susceptibility than any other signaling pathways in more cancer types [9]. PI3K-Akt pathway plays a significant role in a series of cell responses, like cell invasion and migration, which promote the progression of tumor via promoting cell proliferation and inhibiting apoptosis [10, 11]. Genetic variants have been proved to increase the gastric cancer risk. In addition, comparing to gastric cancer patients with lower PIK3R3 expression, higher expression leads to poorer outcome [12]. Inhibition of PI3K promotes inhibition of cell growth and proliferation, induces apoptosis, thereby increasing the chemosensitivity of gastric cancer to drugs [13]. Therefore, the activation of PI3K-Akt pathway is closed related to gastric cancer carcinogenesis and progression.

Furthermore, the extracellular matrix (ECM), acting as a supporting structure, is a substantial component of tissue. The ECM plays a vital role in cell adhesion, intercellular communication and regulates cell differentiation as it provides a crucial source for cell growth, differentiation, proliferation and angiogenic factors which significantly affect cancer biology and progression.

### PPI Network

**Fig. 3:**
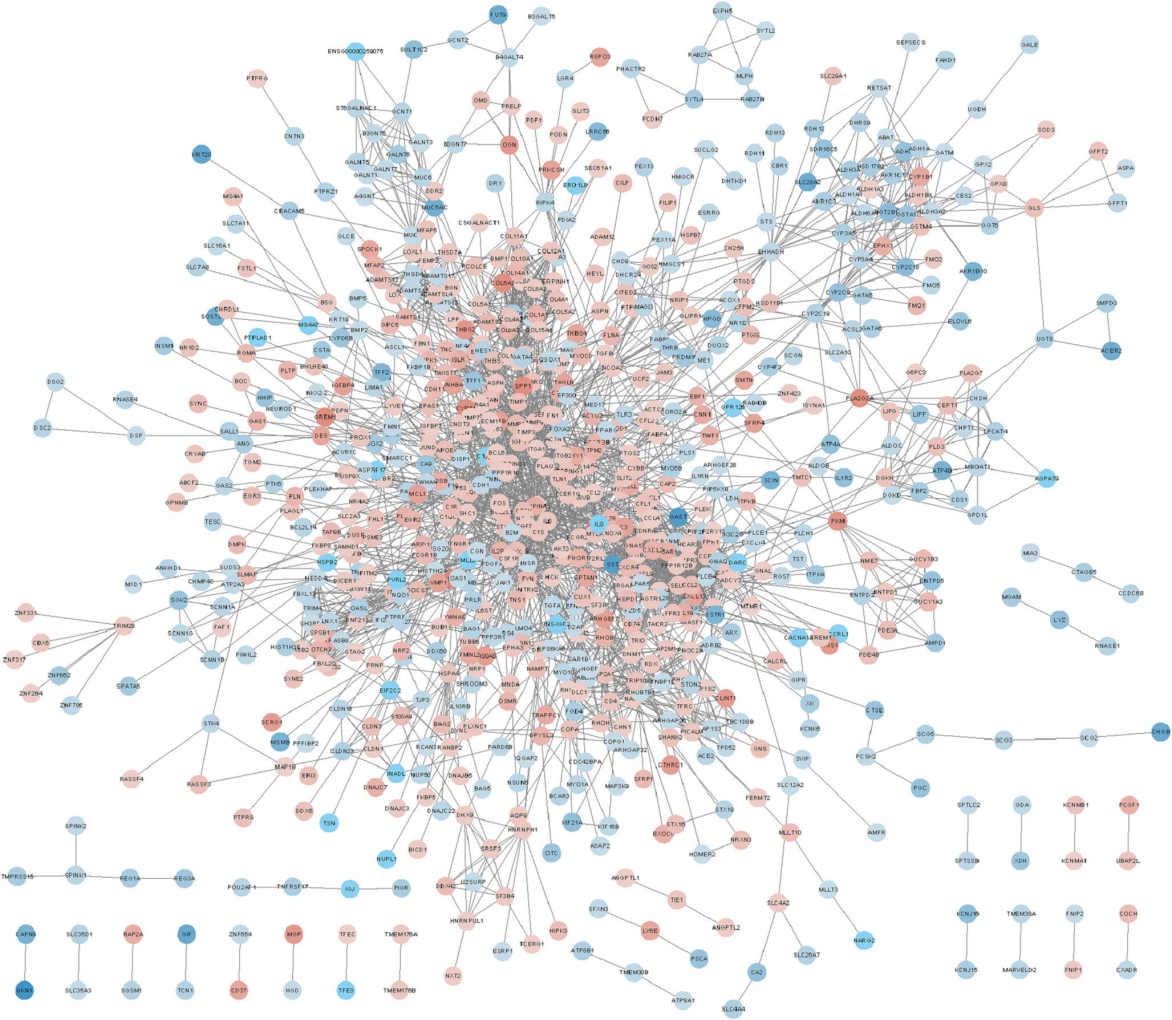
PPI network. Notes: The red points represent up-regulated DEMs, the blue points represent the down-regulated DEMs. The deeper of the color, the larger of the expression changes. Lines between two points represent protein-protein interactions

**Fig. 4:**
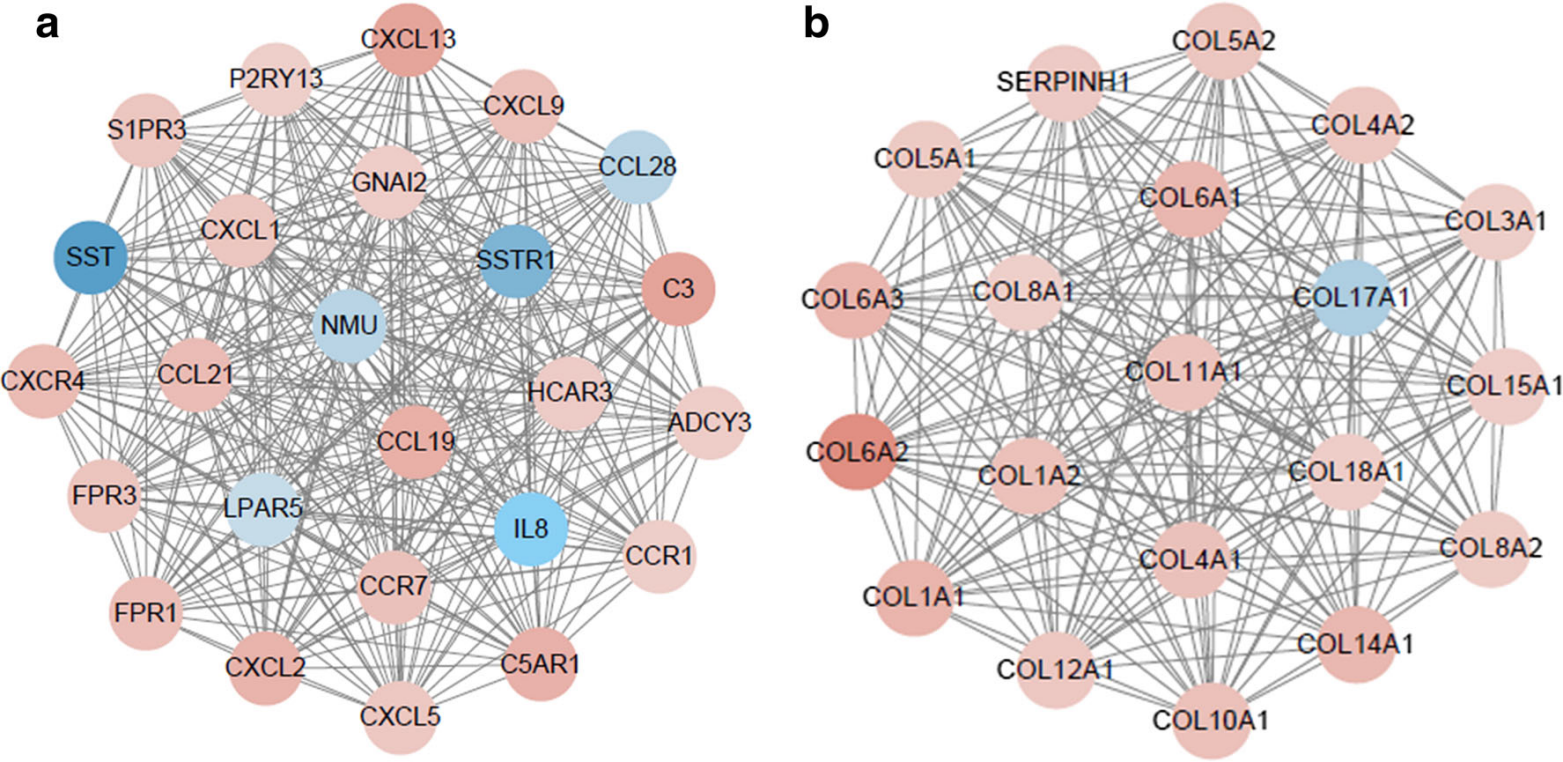
hub subnetworks. Notes: **a** hub subnetworks 1; **b** hub subnetworks 2. The red nodes represent up-regulated DEMs, the blue nodes represent the down-regulated DEMs. The deeper of the color, the larger of the expression changes. Lines between two points represent protein-protein interactions

A total of 3044 protein-protein interactions among 791 DEMs were screened by applying STRING online database (Fig. 3). With the help of MCODE, we identified two hub subnetworks which had the highest score. Most of the genes involved in the two hub subnetworks were members of CXL family and COL family (Fig. 4). In addition, the KEGG pathway enrichment analysis of hub subnetwork 1 showed enrichment in Chemokine signaling pathway, and the genes within hub subnetwork 2 were mainly involved in protein digestion and absorption, ECM-receptor interaction, focal adhesion and PI3K-Akt signaling pathway.

CXCR4, encoding a CXC chemokine receptor specific for stromal cell-derived factor-1, plays an important role in cancer progression and migration [14, 15]. The low expression of CXCR4 is reported to induce apoptosis of human osteosarcoma cells via PI3K-Akt signaling pathway inhibition [16]. Additionally, CXCR4 plays a crucial role in tumor through mediating cell adhesion [17]. Therefore, overexpression of CXCR4 is a critical factor for tumor progression and metastasis via regulating AKT signaling pathway. Peng first examined CCL19 and CCR7 expression in samples from prostate cancer and found that CCL19 and CCR7 were significantly overexpressed in all five prostate cancer cell lines he detected. His study verifies that silencing of CCL19 reduces tumor cell proliferation and suppresses invasion and migration, suggesting that the CCL19/CCR7 axis may mediate the pathogenesis of human prostate tumor [18].

Genes within hub subnetwork 2 are nearly all members of COL family. Previous studies have reported some of these genes. COL1A1 and COL1A2, which encodes the type I collagen. Alterations of type I collagen in the ECM is observed to be related to migration of breast cancer and serves as a poor biomarker of prognosis [19]. Type I collagen has been identified to be over synthesized in the serum of patients with recurrent breast tumor [20]. COL4A1 is not specific for gastric cancer, investigation such genes can provide a better understanding of other cancers.

### ceRNA Network Construction

**Fig. 5:**
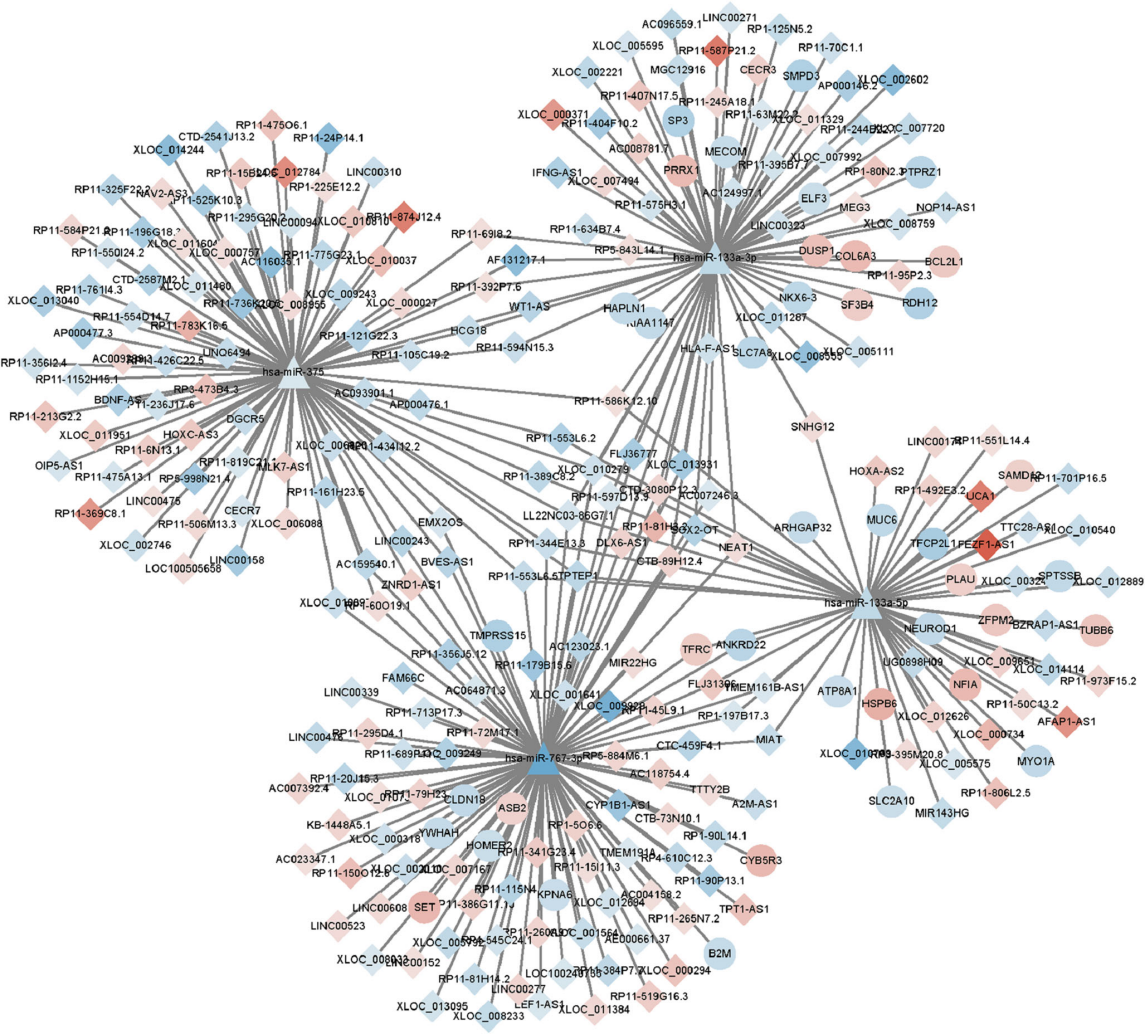
ceRNA network. Notes: The circles represent DEMis, the triangles represent miRNAs and the rhombuses represent lncRNAs. The deeper of the color, the larger of the expression changes. Lines between two points represent protein-protein interactions

**Fig. 6:**
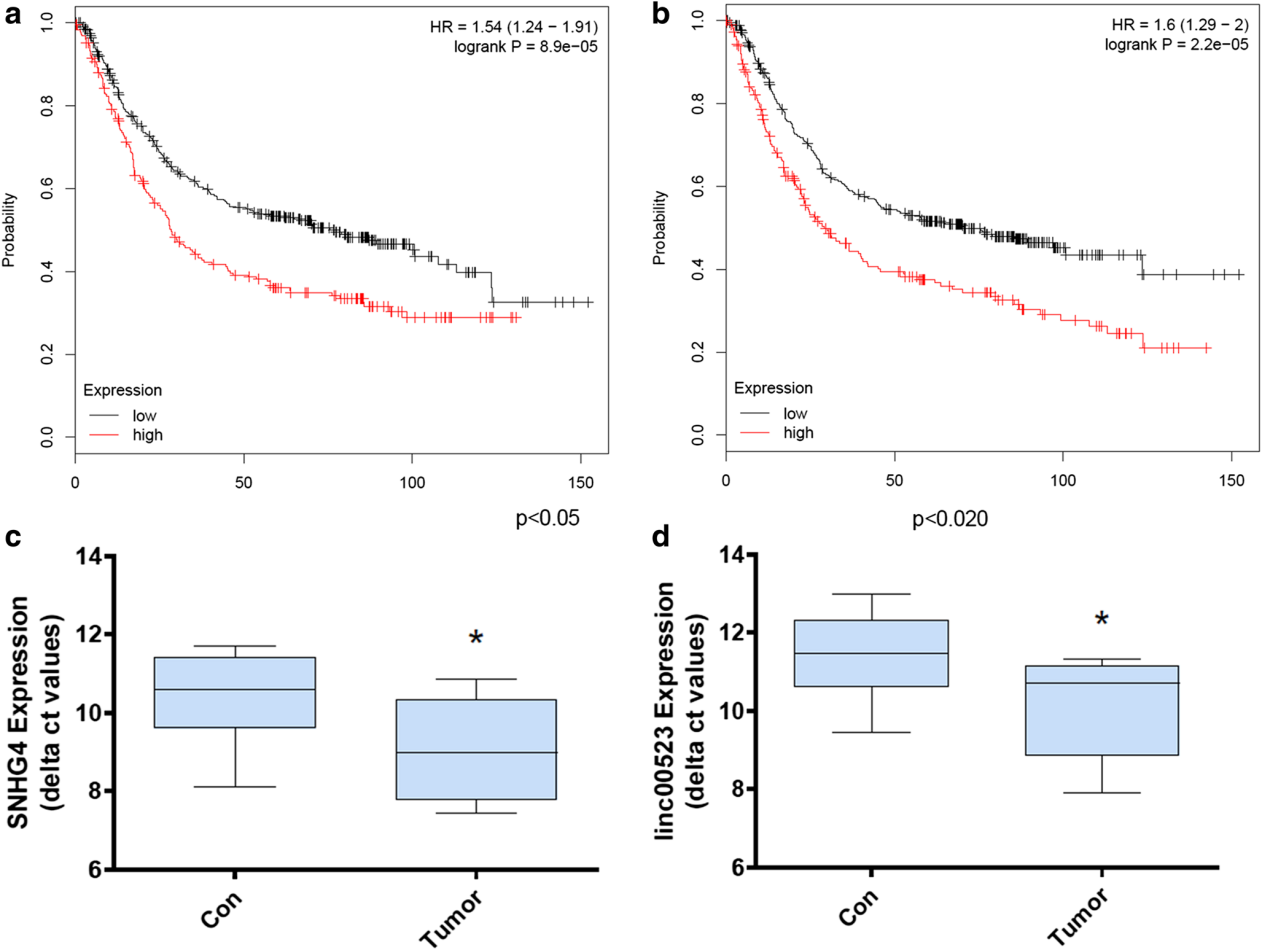
Kaplan–Meier survival analysis and PCR results. Notes: **a** Kaplan–Meier survival analysis of SNHG4; **b** Kaplan–Meier survival analysis of LINC00523; **c** qRT-PCR result for SNHG4; **d** qRT-PCR result for LINC00523. The statistical significance of differences was calculated by the Student’s t test

The mRNAs and lncRNAs predicted by TargetScanHuman and LncBase Predicted v.2 were identified as DEMis target genes. Furthermore, to screen reliable hub genes, the target genes were compared to the DEMs and DELs, only the overlapping genes were selected (Fig. 5). Survival analysis of those lncRNAs were performed. Additionally, SNHG family, acting as a sponge for miRNAs, may regulate gene expression, then promoting tumorigenesis and affecting the immune escape of cancer [21, 22]. We also analyzed the impact SNHG family played on patient outcome. To verify the result, we conducted qRT-PCR with clinical gastric cancer tissues collected. As shown in Fig. 6, the expression of SNHG4 and LINC00523 was up-regulated in gastric cancer. The result suggests that SNHG4 and LINC00523 may indicate a poor prognostic. While the mechanism of SNHG4 and LINC00523 on cancer initiation and progression is remained elusive. And in future, well-designed study may be performed to verify our findings.

## Discussion

In our study, we analyzed the miRNA expression datasets GSE28700 and GSE99415, and 3 overlapping miRNAs were selected as DEMis by comparing their result. Similarly, 1867 DEMs, 966 down-regulated and 901 up-regulated, were screened in GSE54129, and identified 4329 lncRNAs dysregulated in GSE99416, containing 1974 up-regulated and 2355 down-regulated lncRNAs. Furthermore, GO enrichment analysis indicated that DEMs were enriched significantly in extracellular matrix organization, angiogenesis, cell adhesion, endodermal cell differentiation and epithelial cell differentiation at the level of BP. Meanwhile, KEGG pathway enrichment analysis showed that DEMs were mainly involved in focal adhesion, ECM-receptor interaction, Chemical carcinogenesis and Metabolism of xenobiotics by cytochrome P450. In addition, we screened two hub subnetworks by using MCODE app, genes within them were mainly members of CXL family and COL family. Genes within hub subnetwork 1 were mainly involved in Chemokine signaling pathway and Cytokine-cytokine receptor interaction in the level of KEGG pathway, and genes from hub subnetwork 2 showed enrichment in protein digestion and absorption, ECM-receptor interaction, focal adhesion and PI3K-Akt signaling pathway. These enriched pathways provide us insights to reveal the tumorigenesis and progression mechanisms of gastric cancer. Finally, ceRNA was constructed to explore the role of lncRNA played in the gastric cancer initiation and progression. We performed survival analysis for these lncRNAs and differentially expressed long non-coding RNA SNHG family. High expression of LINC00523 and SNHG4 are related to poor outcome. The result of qRT-PCR suggests that their expression is significant elevated in the clinical tumor samples.

Our methods also exist some limitations. Firstly, the microarray applied for DELs screening in the study is not enough. We only analyzed GSE99416 to identify DELs, which consists of 6 gastric cancer tissues and 6 matched non-tumorous adjacent tissues. The small size may introduce false positive. Secondly, the clinical samples enrolled in the validation is also relatively small, a large number of samples warrantees a better reliability.

## Conclusion

We analyzed several microarray datasets from GEO, involving mRNA, miRNA and lncRNA. Functional enrichment analysis, PPI network and ceRNA network were applied to ascertain a comprehensive understanding of the enormous information. Furthermore, survival analysis was performed for some specific lncRNAs, and the expression of them was validated by applying of qRT-RCR. In summary, we hypothesized some novel opinions on carcinogenesis and progression of gastric cancer, which deserves further study.

## Abbreviations

DEMs: Differentially expressed mRNAs
DEMis: Differentially expressed miRNAs
DELs: GO, gene ontology
BP: Biological Process
CC: Cellular Component
MF: Molecular Functions
PPI: Protein-protein interaction
